# Effect of Sodium Gluconate on Properties and Microstructure of Ultra-High-Performance Concrete (UHPC)

**DOI:** 10.3390/ma16093581

**Published:** 2023-05-07

**Authors:** Yonghua Wu, Yibing Yuan, Mengdie Niu, Yufeng Kuang

**Affiliations:** College of Materials Science and Engineering, Xi’an University of Architecture and Technology, Xi’an 710055, China; 13203926241@163.com (Y.Y.); niumengdie@xauat.edu.cn (M.N.); 18821799705@163.com (Y.K.)

**Keywords:** ultra-high-performance concrete (UHPC), sodium gluconate (SG), heat of hydration, competitive adsorption, complexation, cement hydration acceleration period, pore structure

## Abstract

The properties of concrete can be significantly affected by sodium gluconate (SG) at very small dosages. In this paper, the effects of SG on the fluidity, setting time, heat of hydration, and strength of ultra-high-performance concrete (UHPC) were studied. The results show that (1) in the plastic stage, SG inhibited the formation of early ettringite (AFt) and delayed the hydration of tricalcium silicate (C_3_S) and dicalcium silicate (C_2_S). SG increased the initial fluidity of UHPC without decreasing within 1 h. When the SG dosage was ≥0.06%, the slumps at 30 min and 60 min increased slightly. (2) In the setting hardening stage, the addition of SG inhibited the formation of calcium hydroxide (CH), which significantly extended the setting time of UHPC. When the dosage of SG was 0.15%, the initial and final setting times were 5.0 times and 4.5 times that of the blank group, respectively. SG had no obvious effect on the hydration rate of cement in the accelerated period, but the peak hydration temperature of UHPC was increased when the SG dosage was 0.03~0.12%. (3) In the strength development stage, the 1 d and 3 d strength of UHPC decreased significantly with the increase in the SG dosage. However, SG could promote the formation of AFt at the pores and aggregate interface in the later stage, reduce the porosity of cementite, and improve the compressive strength of UHPC in 28 d, 60 d, and 90 d. When the SG dosage was 0.12%, the 90d strength increased by 13%.

## 1. Introduction

Ultra-high-performance concrete (UHPC) is a new cementitious composite material with ultra-high strength, ultra-high durability, and high toughness [1]. The materials used to prepare UHPC mainly include cementitious materials, quartz sand, chemical admixtures, water, etc. Cementitious materials are usually composed of cement, silica fume, fly ash, mineral powder, etc. In addition, UHPC is also mixed with high-strength steel fibers, which can significantly improve the toughness and tensile resistance of concrete [2]. The application of UHPC in engineering can effectively reduce the self-weight of a structure while improving the ductility of the structure, and UHPC with excellent performance is increasingly used in roads, bridges, and long-span projects [3,4]. However, the development and application of UHPC are also limited due to the construction difficulties caused by its rapid setting speed, serious loss of fluidity over time, and the need for higher compressive strength in special projects.

A high-performance superplasticizer is an indispensable component for preparing UHPC [5,6]. In recent years, the rapid development of polycarboxylate superplasticizers (PCEs) has provided technical support for the promotion and application of UHPC. However, the use of PCE alone often cannot meet the combined requirements of UHPC in terms of fluidity, fluidity loss, and setting time. Therefore, it is necessary to compound admixtures such as retarders, air entrainers, and plasticizers, and the application of composite admixtures in UHPC has received increasing attention from researchers [7,8].

Sodium gluconate (SG) is an organic electrolyte with good solubility in water; it belongs to sugars and has a retarding effect when mixed with cement [9,10,11]. However, unlike retarders such as sucrose, citric acid, and sodium tripolyphosphate, the retarding effect of SG is much smaller at the same dosage. The current views on the setting retarding mechanism of SG include the following: (1) Adsorption inhibits the hydration of cement clinker minerals. SG inhibits the hydration of tricalcium silicate (C_3_S), that is, gluconate can inhibit the hydration of C_3_S by adsorbing on the dissolution site on the silicate phase surface [12,13,14], reducing the cumulative hydration heat and hydration rate of cement, prolonging the induction period and retarding setting. (2) Regarding complexation inhibition, another view on the SG retardation mechanism is that the adsorption of SG or the complexation between SG and Ca^2+^ inhibits the formation of ettringite (AFt) [13,15]. (3) Regarding the inhibition of gypsum dissolution, the formation of AFt is related to CaSO_4_·2H_2_O, and SG delays the formation of AFt by preventing the dissolution of CaSO_4_·2H_2_O [14]. (4) Regarding the control calcium hydroxide (CH) theory, the normal precipitation of CH crystals is inhibited by suppressing the continued growth of CH nuclei. At present, it is not clear which of the four roles is the dominant one and how they relate to each other. Further studies have shown that the retarding effect of SG is related to its dosage: when the dosage is low, SG cannot completely neutralize the active dissolution site of C_3_S [13], so there is no gluconic acid available for adsorption on the surface of tricalcium aluminate (C_3_A), and thus the mechanism of SG retardation at low dosages is mainly hindering the nucleation of hydrated calcium silicate and the growth of hydration products [14]. When the dosage is high, the residual SG is completely adsorbed on the surface of C_3_S, and the hydrolysis increases the concentration of Na^+^ and gluconic acid. The remaining gluconic acid prevents the dissolution of CaSO_4_·2H_2_O and is adsorbed on the surface of C_3_A after binding to Ca^2+^, hindering the hydration of C_3_A and the formation of AFt. That is, when the SG dosage is high, it not only delays the hydration of C_3_S but also retards the formation of AFt by preventing the dissolution of CaSO_4_·2H_2_O. Therefore, excessive addition of SG can severely delay the setting time due to the excessive inhibition of hydration. In addition, some scholars [16] believe that SG contains multiple hydroxyl groups that are easy to combine with water molecules to form a stable solvated water film on the surface of cement particles, inhibiting the hydration process of cement and delaying the setting of cement.

Except for its setting retarding effect, SG also has an obvious “auxiliary plasticizing effect”. That is, the water reduction rate of SG itself is very small, but when used together with a superplasticizer (SP), it can significantly increase the water reduction rate of the SP or significantly increase the fluidity of concrete and reduce slump loss [15]. This is because the addition of a small amount of SG can delay the hydration of cement, reduce the amount of the SP’s adsorption, and increase the concentration of the SP in the solution, thereby increasing the fluidity of concrete and reducing slump loss [17,18,19,20,21]. However, the combined use of an SP and retarders has multiple effects [15]. Studies have shown that the auxiliary plasticizing effect of SG is not completely caused by the setting retarding effect [15], such that Tan [22] believes that SG and SPs can enhance steric hindrance by bridging Ca^2+^ and also increase the zeta potential on the surfaces of cement particles, thereby improving the dispersion effect of the SPs. In addition, there is also a competitive adsorption effect between SG and SPs, and the adsorption capacity of an SP will be reduced after adding SG. Previous studies have shown that a small amount of SG can enhance the steric hindrance effect of SPs [23], but excessive SG can hinder the adsorption of SPs [24,25]. According to Tan’s theory [22,26,27], the dispersion capacity of an SP-SG system depends on the amount of SG. When the SG dosage is less than 0.10%, the dispersion enhancement of SG is dominant, while when the SG dosage is greater than 0.10%, the competitive adsorption effect dominates the main advantage [15,25,26]. Li et al. [14] demonstrated that SG improved the initial fluidity of cement paste. The results of Ma et al. [13] showed that the fluidity of cement mortar doped with SG was higher than that without SG, and the maximum dosage of SG in this system was 0.01%. When the dosage was greater than 0.01%, the workability of the cement mortar was not further improved. Regarding liquidity loss, Li et al. [14] showed that the addition of SG can reduce liquidity loss over time. When SG and SP were mixed together, the fluidity loss decreased when the SG dosage was less than 0.03%. A small amount of SG had a significant effect on the liquidity loss of cement at 30 min but had little effect on the liquidity loss after 60 min and 90 min of hydration [13,21].

A trace amount of SG also had a significant enhancement effect on the strength of concrete, which gradually increased with the increase in SG from 0.03% to 0.08% at the same water–cement ratio [28]. The later strength of UHPC was improved via the combined use of SG and SP, but when the SG dosage was greater than 0.1%, the strength of the concrete was seriously reduced due to excessive retardation. The optimal dosage of SG was 0.03~0.07% in cement. With the same water–cement ratio, the strength of concrete can be improved to varying degrees by adding different amounts of naphthalene-based SP and being compounded with different dosages of SG. Each amount of naphthalene-based SP corresponds to an optimal dosage of SG to achieve the highest concrete strength. Moreover, the optimal dosage of SG corresponding to different cement varieties is different, indicating that there are also issues with the suitability of SG and cement. It has been proved that the best compressive strength is obtained at an SG dosage of 0.03% [13]. However, the mechanism by which SG enhances the compressive strength of cement is still unclear. Some scholars have preliminarily analyzed the microscopic mechanism of the use of SG to significantly improve compressive strength via X-ray diffraction analysis (XRD) or scanning electron microscopy (SEM) [13,15]. Ma et al. [13] believe that the surface energy of calcium silicate hydrate (C-S-H) is changed via SG due to adsorption, thereby enhancing the cohesion between C-S-H and improving the compressive strength. Ren et al. [29] found that the formation of AFt could be promoted with small amounts of SG. The rapid formation of AFt was advantageous for the rapid setting and strength development of cement concrete [30]. However, some scholars [13] believe that excessive SG increases the pore size and porosity of cement pastes. Therefore, the influence of SG on the structure of long-aged cement needs to be further studied.

UHPC is a system with an ultra-low water-to-binder ratio, large amounts of cementitious materials, and large amounts of nano-powder. Currently, the mechanism of how SG affects the various properties of UHPC in this system, the extent of its influence, the optimal dosage, and how it affects the hydration leading to a significant increase in strength has been little studied. In addition, existing studies have mostly focused on the influence of SG on one or two stages of concrete plastic, setting hardening, and strength development, while the developments of the three stages of concrete have been shown to be closely linked and interact with each other. Therefore, the systematic analysis of these three stages can comprehensively explain the impact of SG on the cement hydration process. However, a systematic analysis of the entire process of these three stages has not been reported.

**Figure 1:**
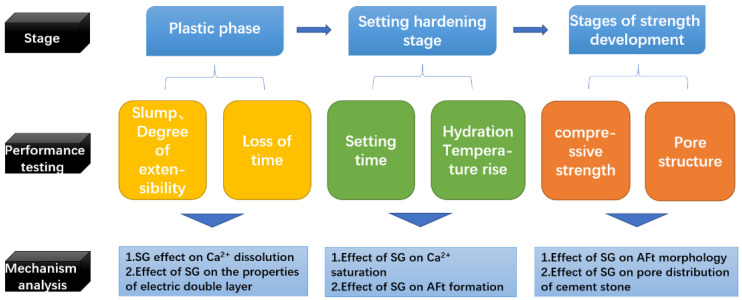
Research roadmap.

In this paper, the effects of SG on the fluidity, setting time, and compressive strength of UHPC are discussed in three sections, and the mechanism of action is analyzed according to the compositions and morphologies of the hydration products, and finally, the mechanism of the effect of SG on UHPC throughout the process from the beginning of water addition to 90 days is discussed, and the research steps are shown in Figure 1.

## 2. Research Significance

Trace amounts of SG can significantly affect the fluidity, setting time, and strength growth of concrete, which has only attracted the attention of a few researchers, and its application in UHPC has not been reported. With a high cementitious material dosage and an ultra-low water-to-binder ratio, the role of SG in UHPC may be more significant. The purpose of this study is to observe the effect of SG on the whole process of the UHPC plasticity stage, setting hardening stage, and strength development stage.

## 3. Experiments

### 3.1. Raw Materials

The cement used in this experiment was Ordinary Portland cement ‘P·O 52.5R’, according to Chinese National Standards GB 175-2007. The specific surface area was 411 m^2^/kg, the chemical composition is shown in Table 1, and the mineral composition of the clinker is shown in Table 2. Silica fume was obtained from Sichuan Langtian Resources Comprehensive Utilization Co., Ltd. (Chengdu, China), and its chemical composition and physical properties are shown in Table 3 and Table 4. The slag powder was S 95 grade slag powder, and the chemical composition of the slag powder was measured with an X-ray fluorescence (XRF) spectrometer, as shown in Table 5.

The particle size of quartz sand was 0.045~1.7 mm, and the chemical composition is shown in Table 6. The coarse aggregate was diorite with a particle size of 5~10 mm and a crushing index of 6.4. The steel fiber was the end-hook-type RS 65/20 steel fiber produced by Zhenqiang High Performance Materials Co., Ltd. (Jiangsu, China). The SP was PCE produced by Sobute New Materials Co., Ltd. (Jiangsu, China). SG was an analytical pure chemical reagent.

### 3.2. Mix Proportion of UHPC

The base mix proportion of UHPC is shown in Table 7, at 30 min the water–binder ratio is 0.16, the steel fiber is the volume content, and the SP dosage is the mass percentage of the cementitious material.

### 3.3. Test Methods

#### 3.3.1. Fluidity and Setting Time

The fluidity of the UHPC mixture, including slump, expansion, and expansion loss over time, was determined in accordance with the relevant provisions of GB/T 50080. The initial and final setting times of UHPC were determined using the penetration resistance method according to the method of T/CECS 864-2021.

#### 3.3.2. Heat of Hydration

The heat of hydration of UHPC was measured according to the direct method in GB/T 12959-2008 by using the proportion and mixing time of the base proportion after removing the coarse aggregate to prepare the samples. According to the calorimeter in a constant temperature environment, the temperature change of the specimen in the calorimeter was determined directly, and the heat of hydration of the cement within 7 d was obtained by calculating the sum of the accumulated and dissipated heat with the calorimeter. The temperature was always maintained at 20 °C during the test, and the temperature change data were recorded continuously for 7 d.

#### 3.3.3. Mechanical Properties

The compressive strength of UHPC was determined according to the relevant provisions of GB/T 50081, using 100 mm × 100 mm × 100 mm cubic specimens.

#### 3.3.4. Microscopic Testing

The hydration products of UHPC containing different doses of SG at different ages were analyzed using XRD. The microstructure of UHPC containing different doses of SG was observed via SEM using a ZEISS Sigma300 (Jena, German), including the morphologies of the hydration products at different ages and the morphologies of the interface areas between the aggregates, steel fibers, and matrix.

The paste was made of SG containing different dosages in the same conditions as the reference mix ratio and mixing time. The prepared samples were poured into plastic cups, and bottles were sealed and stored in a standard curing chamber at a temperature of 20 ± 1 °C and relative humidity of 95 ± 4%. Before performing XRD and SEM, small pieces were soaked in absolute ethanol, and hydration was terminated after 1 d, 3 d, 7 d, and 28 d. The small pieces were then dried to a constant weight in a vacuum drying oven at 45 °C and stored in sealed bags. For XRD analysis, the dried samples were further ground to pass through a 200-mesh sieve. Before SEM testing, the samples were gold-plated at 20 mA for 2 min. XRD analysis was performed with the Bruker D8 Advance model from Germany using CuK radiation in the range of 5–70° with a scanning speed of 10°/min.

## 4. Results and Discussion

### 4.1. Effect of SG on Fluidity in the Plastic Stage of UHPC

#### 4.1.1. UHPC Fluidity Test Results

**Figure 2:**
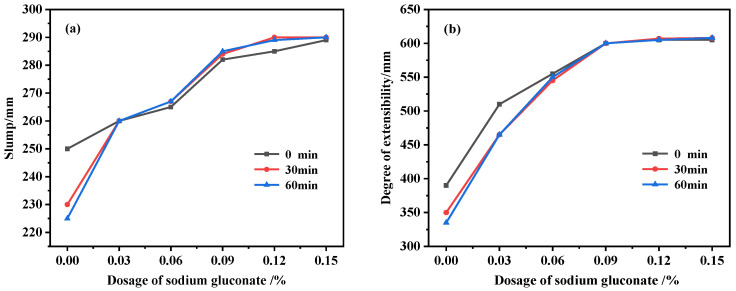
The initial 30 min and 60 min fluidity of UHPC with different dosages of SG. (**a**) Slump; (**b**) degree of extensibility.

Fluidity is an important performance indicator that affects UHPC pouring, which is generally evaluated according to slump and expansion. The initial 30 min and 60 min slumps and expansions of UHPC mixed with SG are shown in Figure 2. With the increase in the SG dosage, UHPC fluidity gradually increased, and it can be concluded from Figure 2a that the initial 30 min and 60 min slumps increased by 16%, 26%, and 29%, respectively. In Figure 2b, it can be seen that the initial 30 min and 60 min expansions increased by 55%, 74%, and 81%, respectively. In addition, the slump and expansion loss over time was obvious in the blank group without SG for 30 min, with an 8% reduction in slump and a 10% reduction in expansion. At 60 min, the time loss was further increased, with further reductions of 2% and 4% in slump and expansion, respectively. When the dosage of the added SG was 0.03%, there was almost no loss of slump at the initial 30 min and 60 min, while there was a slight loss of expansion at 30 min but almost no loss of expansion at 60 min. The 4 groups with 0.06%, 0.09%, 0.12%, and 0.15% SG were added, respectively, and there was almost no time loss within 60 min, and the slump and expansion were almost unchanged or even slightly increased, so it can be determined that SG can significantly reduce the time loss of the UHPC slump. Moreover, UHPC can achieve self-leveling when the dosage of SG is greater than 0.03%, indicating that SG has a significant auxiliary plasticizing effect and slump retention effect on UHPC. This is consistent with the findings of Yu [31] and Hu et al. [32] on cement mortar and ordinary concrete.

#### 4.1.2. XRD Analysis of Hydration Products in the Plastic Stage

**Figure 3:**
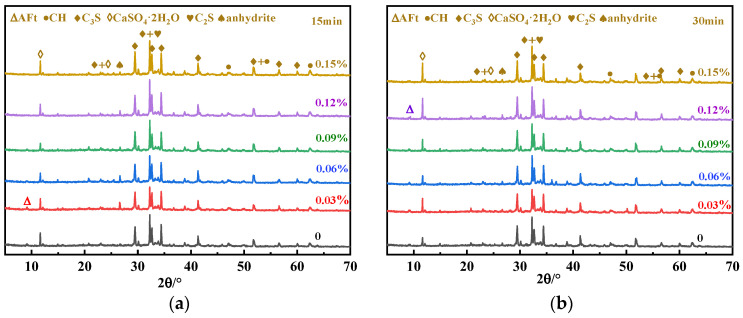
XRD analysis of UHPC hydration products in the plastic stage: (**a**) 15 min; (**b**) 30 min.

UHPC cementitious materials contain a large number of nanoscale particles while using an ultra-low water-to-binder ratio, so there are more SPs. In such a system, the addition of an appropriate amount of SG plays a significant role in the auxiliary plasticizing effect, that is, it significantly improves the initial fluidity while making almost no loss or even a slight increase in the 1h fluidity. There are two reasons for this: one is the retarding effect; the second is competitive adsorption [24,25,33,34]. Tan et al. [22,26,27] believe that the retarding effect of SG reduces the consumption of PCE due to cement hydration, thus improving the dispersion capacity of the PCE-SG system [34]. The XRD plots of the UHPC paste hydrated product at 15 min and 30 min were measured, and the results are shown in Figure 3.

In Figure 3, the diffraction peaks of the hydrated UHPC pastes at 15 min and 30 min are in the same position, indicating that the addition of SG did not change the type of cement hydration products. The higher the SG dosage (0.12% and 0.15%), the higher the diffraction peaks of unhydrated C_3_S and dicalcium silicate (C_2_S), indicating that SG inhibited the hydration of the initial C_3_S and C_2_S. It can also be seen in Figure 3 that a small amount of SG had little effect on the formation of AFt, while an excess of SG significantly inhibited the formation of AFt at 15 min of hydration. This is because the newly formed AFt in the solution adsorbs SG molecules with negative electricity due to its high positive zeta potential. Furthermore, the adsorption of SG molecules on the newly formed AFt crystalline surface inhibits the formation of new crystal planes on the AFt surface. Therefore, the formation rate of the initial AFt was reduced, leading to a decrease in the initial AFt generation [35].

**Figure 4:**
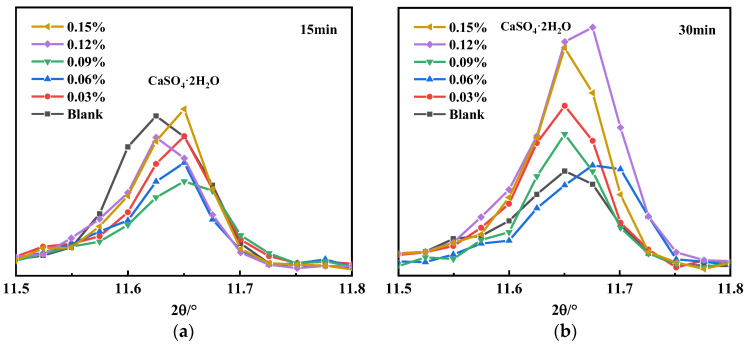
Gypsum diffraction peaks: (**a**) 15 min; (**b**) 30 min.

There are obvious differences in the gypsum diffraction peaks in Figure 3, and the local XRD diffraction peaks of gypsum are shown in Figure 4. The gypsum diffraction peak of the blank sample at 30 min is significantly lower than at 15 min, which indicates that the dissolution of gypsum took place. It can also be seen in Figure 4 that the gypsum diffraction peak decreases first and then increases with the increase in the SG dosage at 15 min, while at 30 min, the gypsum diffraction peak gradually increases with the increase in the SG dosage. This may be because of the two effects of SG of promoting and inhibiting gypsum dissolution: on the one hand, SG can complex with Ca^2+^ in the solution to form calcium gluconate with low solubility, which reduces the concentration of Ca^2+^ in the solution and promotes the dissolution of gypsum due to the homogenic effect. On the other hand, undissolved gypsum in an aqueous solution is positively charged, and SG is an anionic polymer, which adsorbs on the surface of gypsum and hinders the contact between the water and gypsum when the adsorption amount is large, thereby inhibiting the dissolution of gypsum. However, the ion reaction rate in the solution was faster than that of surface adsorption; thus, at 15 min, a small amount of SG mainly promoted the dissolution of gypsum through complexation and solubilization, while at 30 min, the surface adsorption mainly inhibited dissolution, so the solubility of gypsum decreased with the increase in the SG dosage. The gypsum diffraction peak of the SG-doped sample was significantly enhanced at 30 min compared with 15 min, which may be due to the initial AFt formation being inhibited by the composite use of PCE and SG, and the initial dissolved SO_4_^2−^ was not converted to AFt [34]. As the hydration process proceeded, free water decreased, the SO_4_^2−^ concentration increased, and the solubility of CaSO_4_·2H_2_O was lower, so CaSO_4_·2H_2_O crystals were re-formed [29].

**Figure 5:**
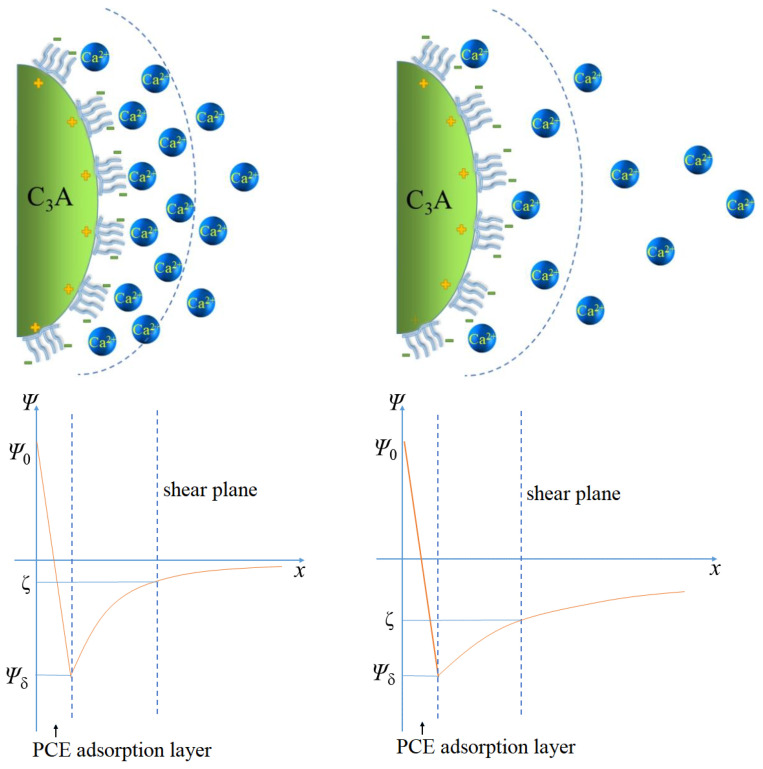
Schematic diagram of Ca^2+^ concentration slurry increasing zeta potential.

In the above XRD analysis, it can be seen that the hydration of cement was inhibited by the addition of SG, thereby reducing the early adsorption capacity of the SP, which is more effective in the adsorption of the SP, thus improving the fluidity. However, in a complex system of multi-component superimposed cement hydration, the mechanism of SG action is not singular but multifaceted: First, after the hydration of cement mixed with the SP, all mineral particles are negatively charged on the surface due to the adsorption of the SP, resulting in electrostatic repulsion and particle dispersion. The positively charged Ca^2+^ in the solution forms an electric double layer with the negatively charged mineral surface, and the concentration of Ca^2+^ is an important factor affecting the zeta potential of the diffusion electric double layer [35]. The magnitude of the zeta potential depends on the counterion concentration within the sliding surface. The more counterions entering the sliding surface, the smaller the zeta potential, and vice versa. Since SG was combined with Ca^2+^ to form water-insoluble calcium gluconate, it reduced the concentration in the solution bilayer, thus increasing the zeta potential of the diffusion electric double layer, which is conducive to improving the electrostatic repulsion between particles and thus increasing the fluidity of the paste (see Figure 5). The second mechanism is competitive adsorption. Compared with PCE, SG is easily soluble in water, and its smaller molecules and high mobility can occupy positively charged active adsorption vacancies on the surface of C_3_A through competitive adsorption [24,25,26,34,35]. Therefore, SG reduces the early adsorption of PCE [24,25,26,34] and increase the remaining amount of PCE in the solution, which is beneficial to the fluidity of cement paste or concrete.

### 4.2. Effect of SG on UHPC Setting and Hardening Process

#### 4.2.1. Effect of SG on UHPC Setting Time

**Figure 6:**
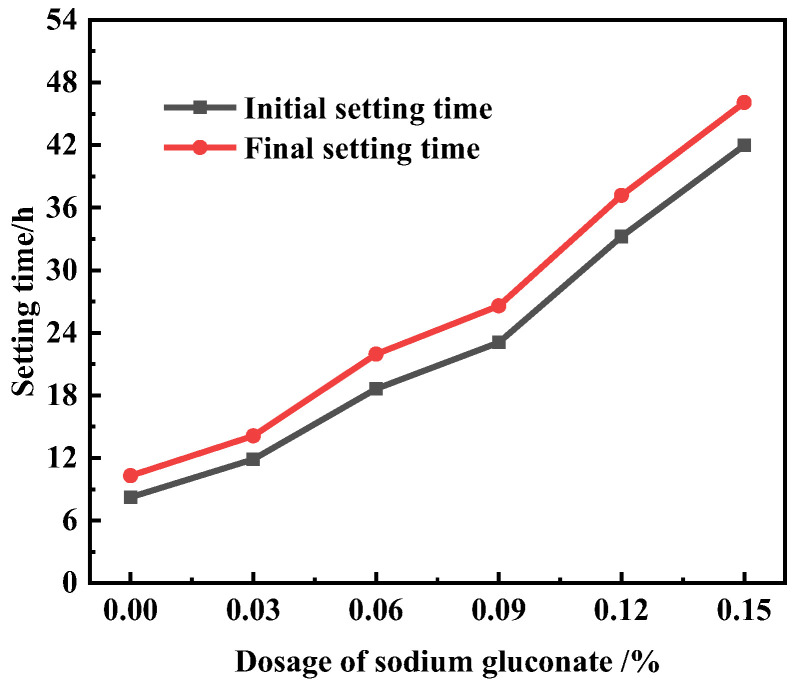
The setting time of UHPC with different dosages of SG.

The results in the previous Section 3.1 show that SG had an impact on the formation of hydration products in the initial stage of cement hydration. The setting and hardening process of cement are closely related to the formation rate and amount of cement hydration products. The effect of SG on the setting time of UHPC is shown in Figure 6. The initial setting time of the blank group was 8 h 14 min, and the final setting time was 10 h 18 min. As the dosage of SG increased, the initial and final setting times of UHPC were significantly prolonged. When the dosage reached 0.15%, the initial and final setting times were 41 h 58 min and 46 h 5 min, which were 5.0 times and 4.5 times those of the blank group, respectively. Compared with the retarding effect of SG in ordinary concrete obtained by other scholars [34,35,36,37], the retarding effect of SG on UHPC is more significant.

**Figure 7:**
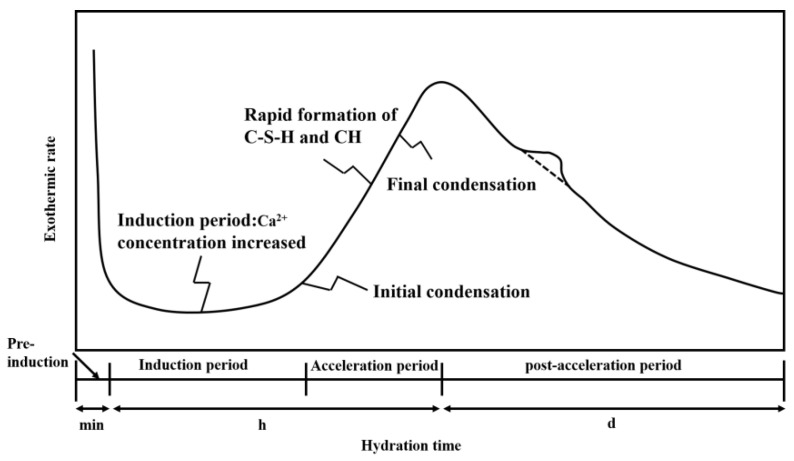
Relationship between cement hydration and heat release process and setting time.

The setting process of cement is closely related to the hydration reaction process of cement. The setting and hardening of cement occur after the induction period, and the initial and final setting times of cement correspond to the beginning and end of the acceleration period in the cement hydration process, respectively, as shown in Figure 7. Although the initial and final setting times were significantly prolonged after adding SG, the difference between the initial and final setting times did not change significantly with the increase in the SG dosage, which indicates that SG significantly prolonged the hydration induction period, while it had little effect on the reaction process during the hydration acceleration period.

#### 4.2.2. Effect of SG on UHPC Heat of Hydration

**Figure 8:**
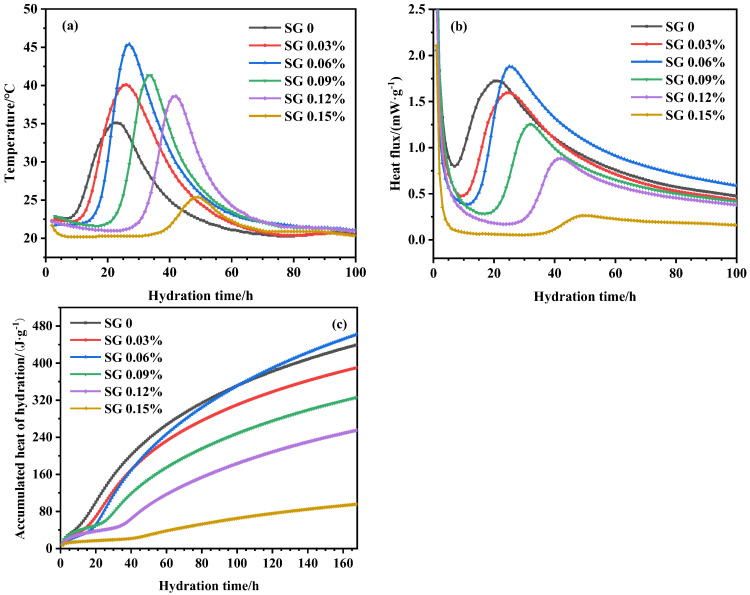
UHPC adiabatic temperature rise, heat flow, and accumulated heat of hydration with different dosages of SG. (**a**) Adiabatic temperature rise; (**b**) heat flow; (**c**) accumulated hydration heat.

The changes in the temperature, heat flow, and cumulative hydration heat of UHPC with different SG dosages are shown in Figure 8. The heat flow represents the hydration rate of cement. As can be seen in Figure 8, the hydration kinetics of UHPC was significantly altered by the incorporation of SG. With the increase in the SG dosage, the hydration induction period of UHPC was significantly prolonged, indicating that SG retarded the hydration of C_3_S. The acceleration phase started with a delay, which is consistent with the variation in the initial setting time in Figure 6. As also shown in Figure 8, the addition of SG significantly changed the peak value of the UHPC hydration temperature. With the increase in the SG dosage, the hydration temperature peak of UHPC increased first and then decreased, indicating that different dosages of SG had different effects on the hydration heat release of UHPC. The experimental results of this study are basically consistent with the results of SG in ordinary concrete [31,36].

The slope of the acceleration period curve in Figure 8 was calculated via numerical fitting, and the slopes of the acceleration period were 0.12, 0.14, 0.20, 0.10, 0.09, and 0.02 for SG dosages of 0%, 0.03%, 0.06%, 0.09%, 0.12%, and 0.15%, respectively. It shows that when the SG dosage is <0.06%, the slope increases with the increase in the dosage, indicating that SG promoted a hydration acceleration period within this dosage range. When the SG dosage is >0.06%, the slope decreases with the increase in the SG dosage, which indicates that SG inhibited the hydration acceleration period within this dosage range. When the SG dosage is 0.06%, the hydration temperature peak reaches the maximum, and SG had the greatest promotion effect on the hydration acceleration period at this dosage. When the SG dosage reaches 0.15%, the hydration exothermic peak decreases significantly, indicating that a high SG dosage excessively inhibits the early hydration of UHPC [38] and is not conducive to the development of the early strength of UHPC.

The variation in the accumulated hydration heat with hydration time is shown in Figure 8c. As the early dosage of SG increased, the early accumulated heat of UHPC significantly decreased. After the acceleration period, the accumulated heat of hydration of UHPC mixed with SG accelerated and increased faster, whereby the accumulated heat of hydration of UHPC with 0.06% SG was the fastest growth rate, catching up and surpassing the blank group after 90 h. However, the cumulative hydration heat grew slowly when the SG dosage was 0.15%. This indicates that an appropriate amount of SG promotes the early hydration of UHPC, but an excessive amount significantly inhibits the early hydration reaction of UHPC [38].

**Figure 9:**
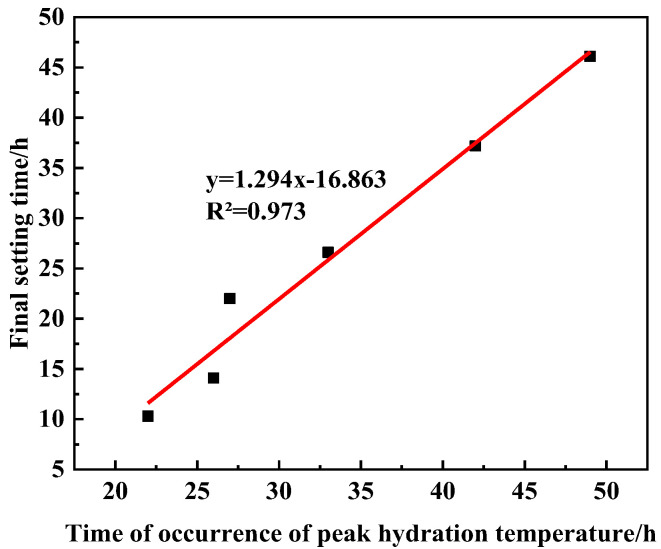
Relationship between the occurrence time of hydration temperature peak and the final setting time.

Comparing Figure 6 and Figure 8, it can be seen that there is a significant correlation between the UHPC hydration temperature change and setting time, and the relationship between the occurrence time of hydration temperature peak and the final setting time is shown in Figure 9. It can be seen in Figure 9 that the occurrence time of the UHPC hydration temperature peak was linearly correlated with the final setting time, and the correlation coefficient is R = 0.973. This also shows that the setting process of cement was closely related to the formation process of hydration products of cement.

#### 4.2.3. Setting and Hardening Stage XRD and SEM Test Results

**Figure 10:**
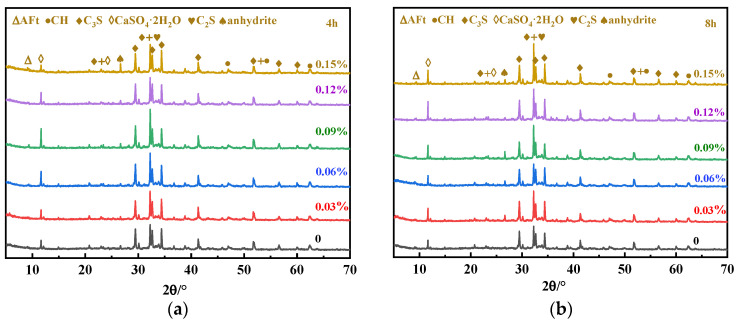

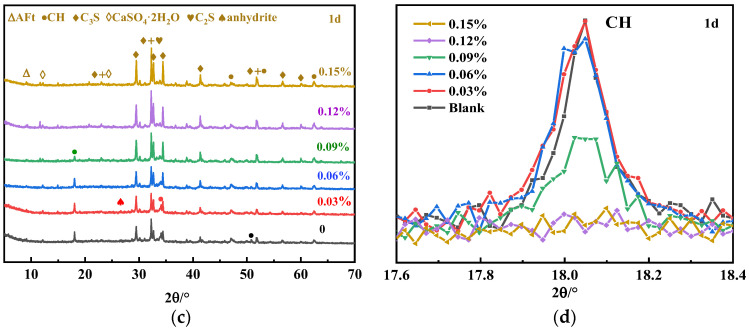
UHPC slurry doped with SG 4 h, 8 h, and 1 d XRD plots: (**a**) 4 h; (**b**) 8 h; (**c**) 1 d; and (**d**) 1 d CH local characteristic peaks.

**Figure 11:**
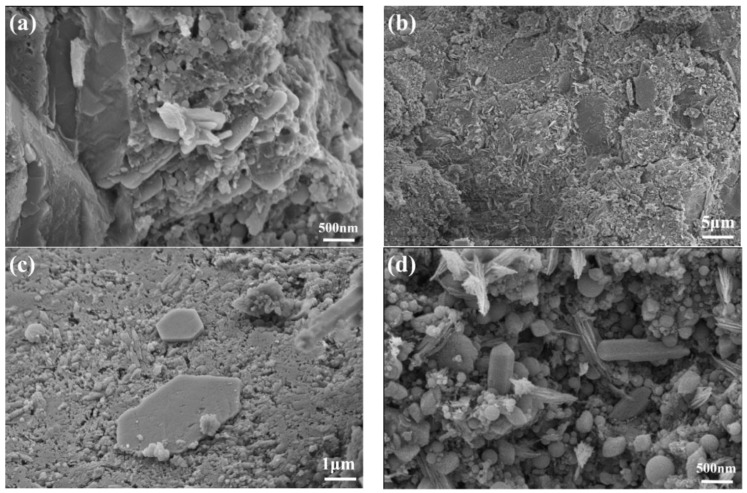
CH scanning electron microscopy of SG-doped UHPC slurry after hydration for 1 d. (**a**) Without SG; (**b**) with 0.03% SG; (**c**) with 0.06% SG; (**d**) with 0.09% SG.

The XRD patterns of the UHPC paste at 4 h, 8 h, and 1 d are shown in Figure 10. It can be seen that the CH diffraction peaks are not obvious at 4 h and 8 h, while significant CH diffraction peaks appear at 1 d for the blank sample and the samples with SG dosages of 0.03%, 0.06%, and 0.09%. In Figure 6, it can be seen that the blank sample and the samples with 0.03% and 0.06% SG additions are finalized at 1 d, and the sample with an SG dosage of 0.09% has initially set, but the samples with SG dosages of 0.12% and 0.15% have not yet reached initial setting at 1 d. This shows that obvious CH crystals were formed after the initial setting of UHPC. The SEM images in Figure 11 also confirm the significant CH formation in the blank sample and the samples with SG dosages of 0.03%, 0.06%, and 0.09% at 1 d of hydration.

The important reason for cement hydration to enter the accelerated period after the induction period is that the Ca^2+^ concentration reaches supersaturation and is able to form CH, which promotes the large-scale formation of C-S-H gels [36]. SG is adsorbed on the surface of C_3_A in cement and can inhibit the dissolution of Ca^2+^. Meanwhile, the concentration of Ca^2+^ in the solution is reduced due to the formation of insoluble calcium gluconate. These two effects prolonged the time for Ca^2+^ to reach supersaturation, thus delaying the initial setting time of cement [16,31,36]. When SG was consumed, the concentration of Ca^2+^ increased continuously and reached supersaturation, which eventually promoted the crystallization of CH and the mass production of C-S-H gels, and the cement’s initial setting. Since SG mainly interacted with C_3_A and had little effect on C-S-H, thus SG did not affect the hydration rate during the acceleration period. On the other hand, the solubility of calcium gluconate was very small at room temperature, but its solubility increases significantly with an increase in temperature. Therefore, during the acceleration period as hydration proceeded, the temperature increased, and calcium gluconate continued to dissolve, causing a rapid increase in the Ca^2+^ concentration in the solution, thereby promoting hydration and increasing the peak hydration temperature. However, when SG was excessive (such as at 0.15%), it not only adsorbed on the surface of C_3_A but also heavily adsorbed on the surface of positively charged AFt. The growth of AFt crystals in the encapsulation layer formed during the induction period of cement, which leads to the rupture of the encapsulation layer, was an important factor affecting the hydration rate of cement during the acceleration period. Therefore, the excess SG significantly affected the hydration rate of cement during the acceleration period and significantly reduced the hydration exothermic temperature and early accumulated hydration heat release [16,31,36].

### 4.3. Effect of SG on UHPC Strength Development and Pore Structure

#### 4.3.1. Effect of SG on Compressive Strength of UHPC

**Figure 12:**
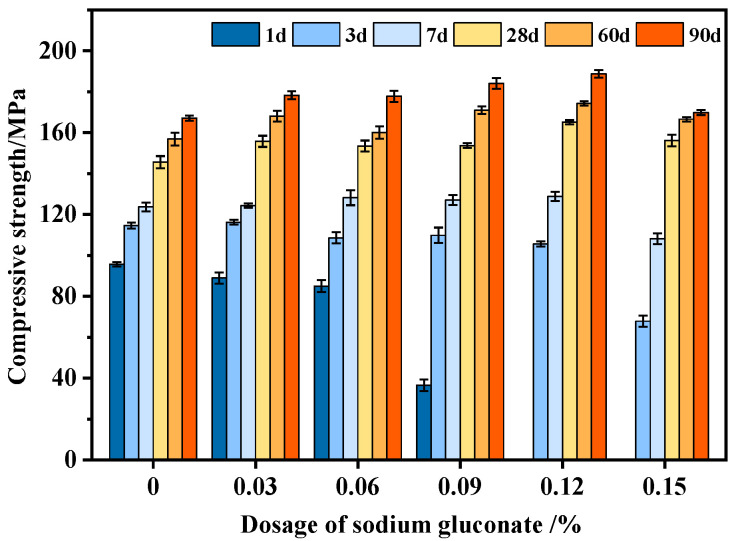
The compressive strength of UHPC with different dosages of SG.

The speed of concrete setting and hardening significantly affects the early strength of concrete, but it generally has little effect on the later strength. Figure 12 shows the standard curing compressive strength of UHPC with different SG dosages. When the SG dosage was <0.06%, the 1 d compressive strength of UHPC decreased slightly with the increase in the SG dosage. When the SG dosage was 0.03% and 0.06%, the strength decreased by 6% and 11%, respectively. When the dosage of SG was >0.06%, the compressive strength was significantly reduced, and when the dosage was ≥0.12%, UHPC was not completely set and had no strength at 1 d. At 3 d and 7 d, the compressive strength of UHPC decreased significantly with the increase in the SG dosage, while at 28 d, 60 d, and 90 d, the compressive strength of UHPC was higher than that of the blank group. In summary, at 28 d, 60 d, and 90 d, the compressive strength of UHPC was enhanced when the dosage of SG was in the range of 0.03~0.12%. When the SG dosage was 0.12%, the maximum enhancement of 90 d strength was 13%. While the compressive strength of concrete at the 60 d age was the largest, the corresponding optimal SG dosage was 0.05~0.07% in the research by Hu [32]. The optimal SG dosage in this test study was higher (0.12%) because the test duration was longer (90 d), and there were more cementitious materials.

**Figure 13:**
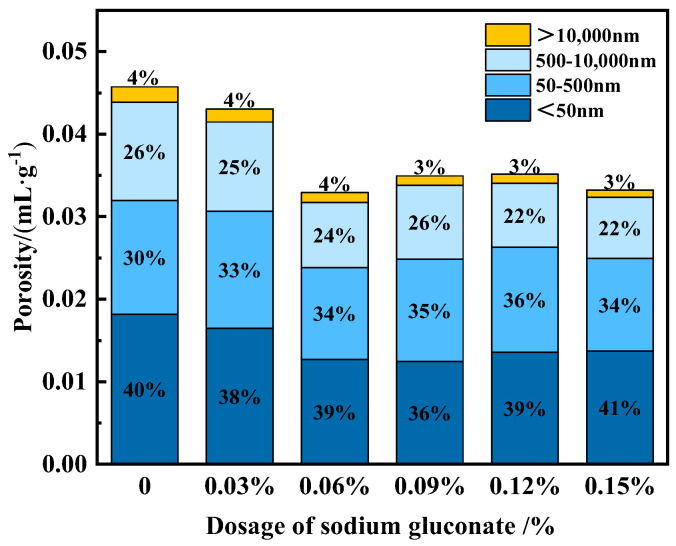
Effect of SG dosage on porosity and pore distribution of UHPC.

**Figure 14:**
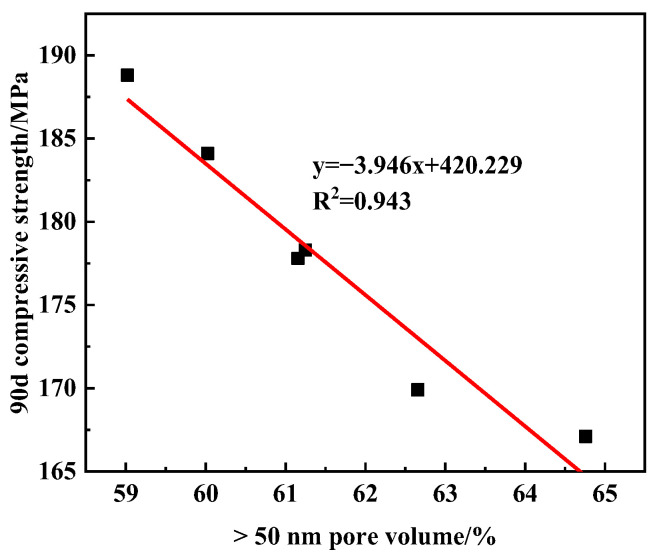
Relationship between pore volume greater than 50 nm and compressive strength.

Porosity and pore structure are important factors affecting the strength of concrete. The porosity and pore distribution of UHPC with different SG dosages at 90 days were tested using the mercury pressure method, and the results are shown in Figure 13. It can be seen that the porosity of the sample with SG was smaller than that of the blank sample. Moreover, when the SG dosage was 0.06%, 0.09%, 0.12%, and 0.15%, the total porosity of the samples was significantly reduced, and the number of harmful pores greater than 50 nm was also reduced. The number of harmful pore volumes greater than 50 nm significantly affects the strength of concrete, and the correlation between harmful pore volumes greater than 50 nm and the compressive strength of 90 d concrete is shown in Figure 14. In Figure 14, it can be seen that the smaller the volume of harmful pores greater than 50 nm, the higher the compressive strength of concrete. Moreover, there is a significant linear negative correlation between the two, with a correlation coefficient of R = 0.943. This is an important reason for the improvement in the strength of UHPC doped with SG.

#### 4.3.2. XRD Test Results at Different Ages

**Figure 15:**
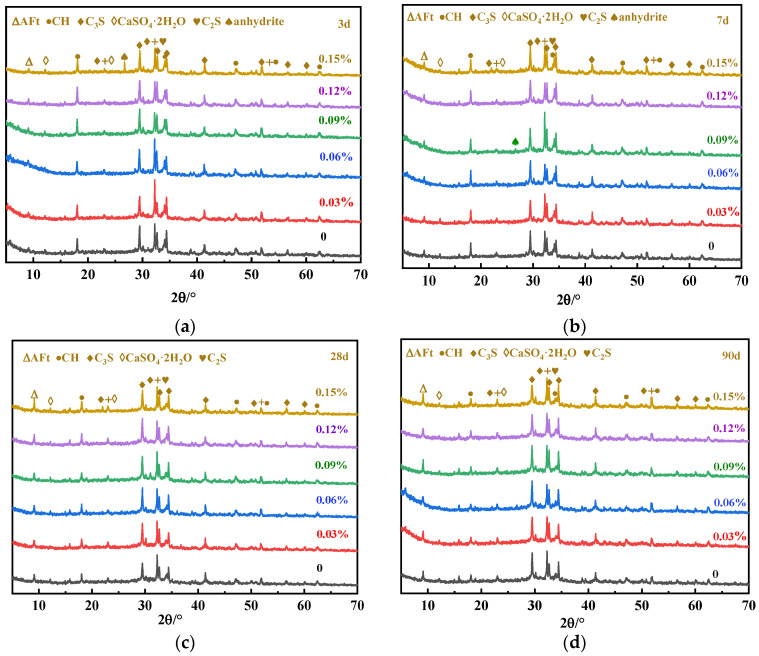
XRD diagrams of cementitious materials with different amounts of SG at different hydration ages: (**a**) 3 d; (**b**) 7 d; (**c**) 28 d; and (**d**) 90 d.

The XRD patterns of UHPC with different SG doping at 3 d, 7 d, 28 d, and 90 d are shown in Figure 15.

**Figure 16:**
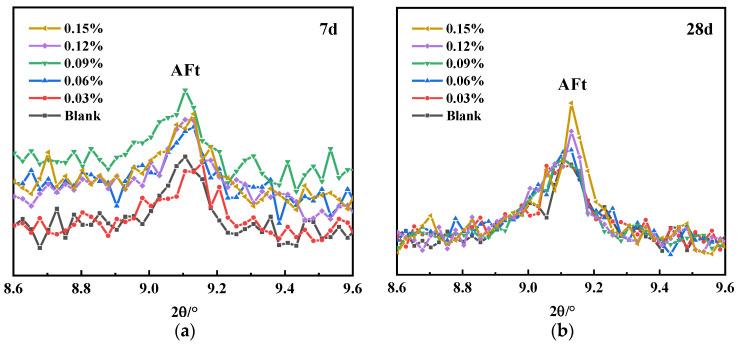

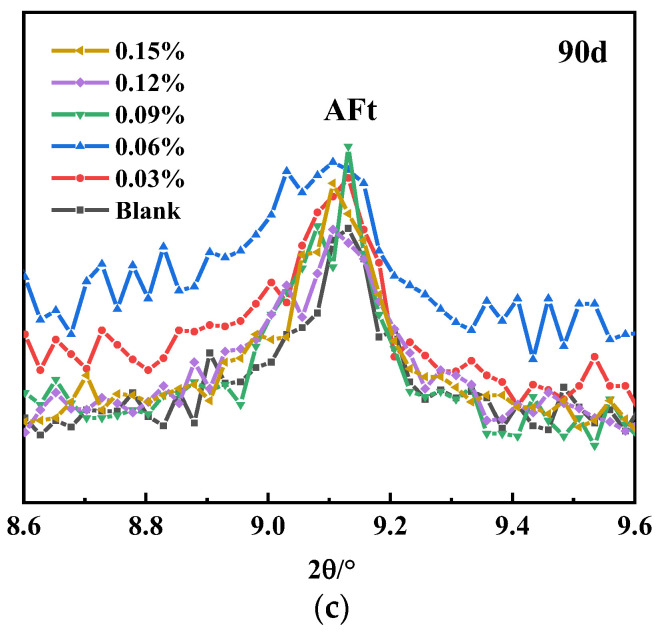
AFt local diffraction patterns of hydration at 7 d, 28 d, and 90 d: (**a**) 7 d; (**b**) 28 d; (**c**) 90 d.

As can be seen in Figure 15, there are obvious CH and AFt diffraction peaks in the XRD plots at 3 d, 7 d, 28 d, and 90 d. The CH peak gradually weakens with the curing age, while the AFt peak gradually strengthens, and, especially for SG-doped samples, the increase in the AFt peaks is more obvious. Figure 16 shows the local diffraction patterns of AFt at 7 d, 28 d, and 90 d. It can be seen that the addition of SG increases the diffraction peak strength of AFt at all three ages, indicating an increase in the production of AFt. Figure 16c also shows that the AFt diffraction peak is widened by the addition of SG, indicating that the crystalline morphology of AFt changed.

#### 4.3.3. SEM Test Results at Different Ages

**Figure 17:**
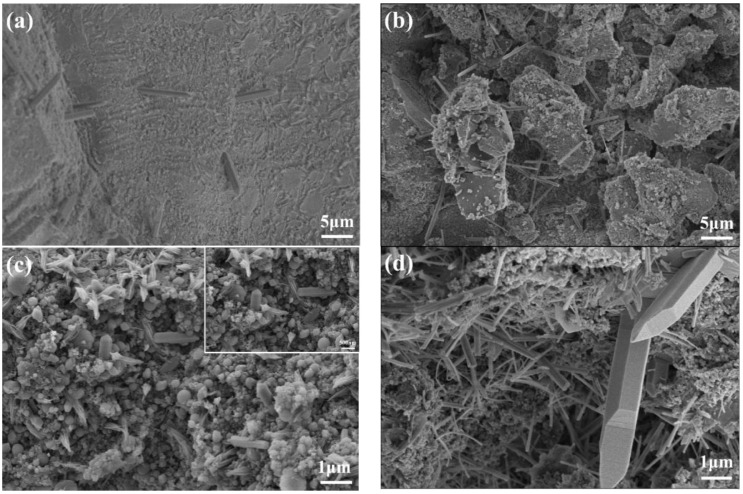

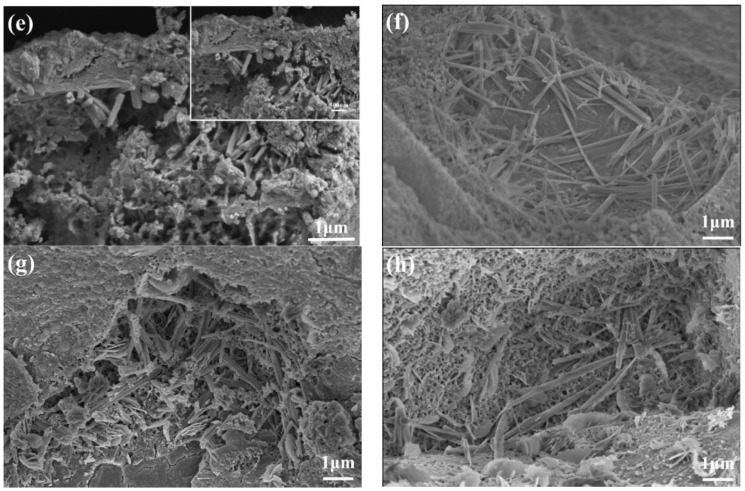
SEM images of AFt of hydration products in UHPC slurries at different ages. (**a**) Without SG (1 d); (**b**) with 0.06% SG (1 d); (**c**) with 0.09% SG (1 d); (**d**) with 0.03% SG (7 d); (**e**) with 0.09% SG (28 d); (**f**) with 0.03% SG (90 d); (**g**) with 0.12% SG (90 d); (**h**) with 0.15% SG (90 d).

The reason for the ability of SG to significantly increase the strength of UHPC can be observed in the SEM images of the hydration products at different ages. Figure 17 shows the SEM images of AFt in UHPC with different SG dosages at different hydration ages. As can be seen in Figure 17a, a small amount of AFt formed at 1 day for the blank sample, which was complete and coarsely crystalline. In Figure 17b, the AFt crystals become significantly finer after adding 0.06% SG. In Figure 17c, the AFt crystals of samples doped with 0.06% became shorter, but the number increased. As hydration continued, more alumina formed in the pores of the cement paste in the form of elongated needle rods, and the number of AFt crystals increased significantly and staggered symbiotically, as shown in Figure 17d–h. Due to AFt mostly existing in the pores, aggregate interface, and fiber interface where the water–cement ratio is large, it filled the pores and significantly improved the compactness of the concrete, thus significantly increasing the strength.

**Figure 18:**
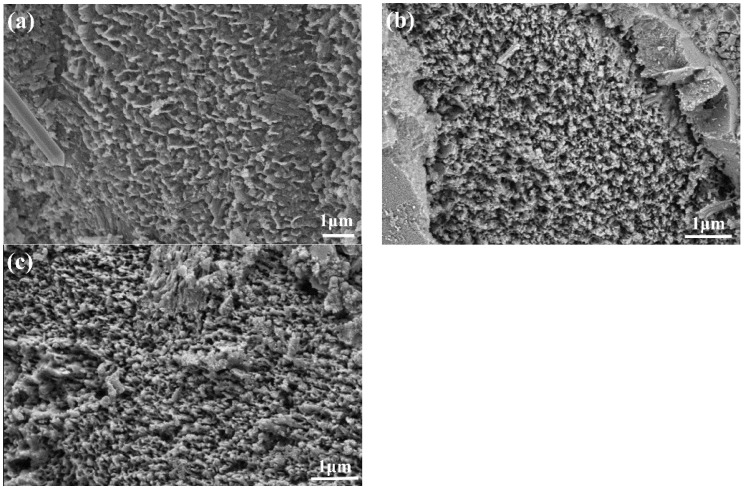
SEM images of the hydration product C-S-H of UHPC with different dosages of SG at 28 d age (**a**) without SG; (**b**) with 0.06% SG; and (**c**) with 0.12% SG.

**Figure 19:**
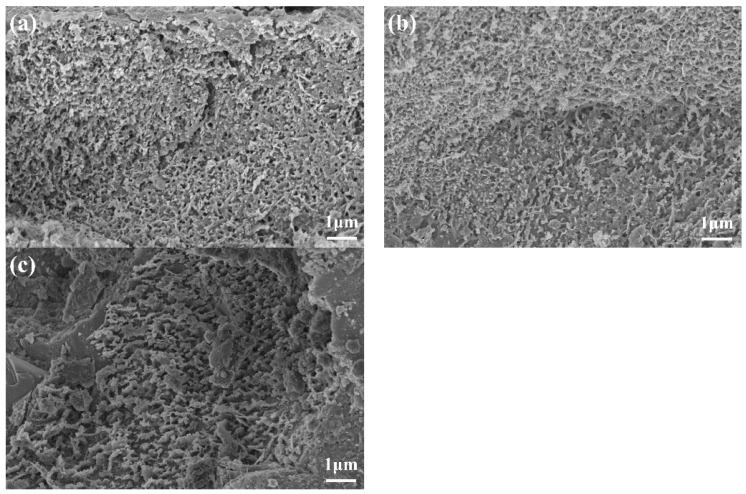
SEM images of the hydration product C-S-H of UHPC with different dosages of SG at 90 d age (**a**) without SG; (**b**) with 0.06% SG; and (**c**) with 0.12% SG.

Figure 18 and Figure 19 show SEM images of C-S-H in UHPC with different SG dosages at 28 d and 90 d. It can be seen that the intertwined C-S-H gel particles formed a network structure with a proportion of flocculated gels, relatively, with the increase in age. However, the microscopic morphologies of the C-S-H gels of samples with different SG dosages had little difference at different ages. This shows that SG has little effect on C-S-H gels.

### 4.4. Discussion

#### 4.4.1. Analysis of the Influence of SG on the Whole Process of Cement Hydration

**Figure 20:**
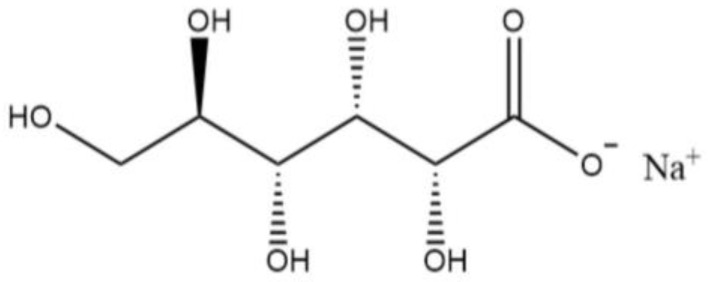
SG molecular structure.

The previous experimental results show that SG has a significant effect on the fluidity, hydration, setting and hardening, and mechanical properties of cement. Carbohydrates are mostly high-efficiency retarders but are unlike glucose and sucrose, whose molecular structures are mostly cyclic structures. SG is a ring-opening structure, as shown in Figure 20. After the dissolution of Na^+^ ions in water with a negatively charged carboxylic acid at one end, SG was able to absorb with positively charged adsorption vacancy on the surfaces of cement particles. The alkyl backbone of SG contains five hydroxyl groups, which can form a solvated water film layer via hydrogen bonding with water molecules. In addition, SG is easily soluble in water, while the solubility of calcium gluconate is very low at room temperature but increases significantly at high temperatures. Therefore, adding SG to cement has the adsorption coverage function of inhibiting hydration, competitive adsorption, and complexation to stabilize Ca^2+^ [24,25,26,33,34].

During the plastic stage of the UHPC paste, SG inhibited the hydration of initial C_3_S and C_2_S and the formation of AFt due to adsorption coverage, while the competitive adsorption effect of SG resulted in the presence of more PCE in the solution. These aspects resulted in a significant increase in the initial fluidity of the UHPC paste doped with SG and a reduction in the fluidity loss [24,25,26,33,34].

During the setting and hardening stage of UHPC, the stabilizing Ca^2+^ effect of SG complexation and the continuation of the previous adsorption covering effect significantly prolonged the time for Ca^2+^ to reach saturation, thereby greatly extending the initial setting time of UHPC. However, due to the increase in the hydration temperature of UHPC during the accelerated period, the solubility of calcium gluconate increased and Ca^2+^ was re-released. Therefore, there was no significant effect of SG on the hydration rate during the acceleration period.

During the strength development stage of UHPC mixed with SG, due to the slower hydration in the early stage, the hydration products generated in the early stage were more uniformly distributed. More AFt with finer grains and staggered symbiotics were generated in the pores and interfaces, which improved the pore structure of the UHPC matrix, thus improving the compressive strength of UHPC.

#### 4.4.2. Selection of SG Dosage

With the increase in the SG dosage, the initial fluidity and fluidity retention continued to increase, and the initial and final setting times continued to increase. However, the early strength (1 d and 3 d) gradually decreased with the increase in the SG dosage, while the later strength (28 d, 60 d, and 90 d) increased with the increase in the SG dosage. The greater the SG dosage, the longer the enhancement effect takes to appear. Considering the influence of fluidity, setting time, and strength, the amount of SG in UHPC should be 0.03~0.09%, and too much SG leads to severe retardation and increased cost.

#### 4.4.3. Study Limitations and Recommendations

The amount of SG added to UHPC was very small, but it had a significant effect on its fluidity, setting time, and strength. The effects of SG on the composition, morphology, hydration heat, and pore structure of hydration products were studied in this paper. However, due to the complex composition of UHPC and the small amount of SG, many differences cannot be well characterized with existing research methods, especially since the existence of admixtures in hardened concrete has not been reported. In subsequent studies, new means can be used to study the existence of forms of admixtures in the hardened matrix.

SG is an organic electrolyte that exists in the form of ions in cement solutions. When cement is mixed with various electrolytes (such as sulfate, chloride, etc.), it significantly affects the ion concentration in the solution and affect the effectiveness of SG, and this effect needs to be further studied. In addition, UHPC has the characteristics of high toughness and ultra-high durability, and the effect of SG on the toughness, shrinkage performance, and durability of UHPC should be researched in the future.

## 5. Conclusions

In this paper, the influences of SG on the fluidity, setting times, hydration heat, and compressive strength of UHPC were studied, and the mechanism of SG was preliminary analyzed through XRD, SEM, and mercury pressure, and the main conclusions are as follows:

(1) The fluidity loss of UHPC without SG was significant within 30 min, at approximately 10%. It was further reduced at 60 min, with a loss of approximately 4% at 60 min. When the initial fluidity of UHPC was 0.15%, the initial slump and expansion of UHPC increased by 15.6% and 55.1%, respectively, and the fluidity did not decrease or increased slightly within 1 h.
(2) The addition of SG significantly prolonged the initial and final setting times of UHPC but had little effect on the interval between the initial and the final setting times. SG inhibited the dissolution of gypsum in cement and delayed the formation of AFt in the early stage of hydration. SG can also complex with Ca^2+^ to generate insoluble calcium gluconate, inhibit the hydration of C_3_S and C_2_S, prolong the time for Ca^2+^ to reach saturation, prolong the induction period, and thus delay the setting time of UHPC.
(3) SG significantly affected the hydration kinetics of UHPC but had no obvious effect on the hydration rate of cement during the acceleration period. The addition of 0.06~0.12% SG reduced the peak hydration temperature and the heat of hydration of UHPC.
(4) When the SG dosage exceeded 0.09%, the 1 d and 3 d strengths of UHPC decreased significantly, but the strength from 7 d to the later stage was not affected and could significantly exceed that of the blank sample. When the SG dosage reached 0.12%, the compressive strength at 90 d increased by 13.0% compared with the blank group. SG causes more AFt to form in the pores in the later stage, reduces the porosity of UHPC, improves the pore structure, and thus effectively enhances the strength of UHPC.

## Figures and Tables

**Table 1:** Chemical composition of cement (%).

CaO	SiO_2_	SO_3_	Fe_2_O_3_	Al_2_O_3_	MgO	K_2_O	Na_2_O	TiO_2_	MnO	P_2_O_5_	Sum
67.67	17.12	4.24	4.15	3.06	1.73	0.732	0.399	0.254	0.0995	0.0824	99.54

**Table 2:** Mineral composition of cement clinker.

C_3_S	C_2_S	C_3_A	C_4_AF
56.06	21.61	6.25	11.3

**Table 3:** Chemical composition of silica fume (%).

SiO_2_	SO_3_	K_2_O	CaO	MgO	P_2_O_5_	Na_2_O	Al_2_O_3_	Fe_2_O_3_	MnO	TiO_2_	Sum
96.68	0.873	0.701	0.489	0.448	0.327	0.127	0.126	0.0849	0.0063	0.0031	99.87

**Table 4:** Physical properties of silica fume.

Content of Cl^−^ (%)	Specific Surface Area (m^2^·kg^−1^)	Water Content (%)	Loss (%)	Activation Index (%)	Density (kg·m^−3^)
0.02	18991	0.94	3.13	115	310

**Table 5:** Chemical composition of slag powder (%).

CaO	SiO_2_	Al_2_O_3_	MgO	SO_3_	TiO_2_	Na_2_O	K_2_O	Fe_2_O_3_	MnO	P_2_O_5_	Sum
44.78	28.83	12.71	8.05	1.50	1.39	0.744	0.522	0.408	0.360	0.0194	99.31

**Table 6:** Chemical composition of quartz sand (%).

Al_2_O_3_	SiO_2_	Fe_2_O_3_	CaO	MgO	K_2_O	Na_2_O	TiO_2_	Loss (1025 °C)
0.29	99.31	0.011	0.02	0.02	0.06	<0.01	0.07	0.13

**Table 7:** Mix proportion of UHPC.

Water-to-Binder Ratio	Cement (kg·m^−3^)	Silica Fume (kg·m^−3^)	Slag Powder (kg·m^−3^)	Quartz Sand (kg·m^−3^)	Coarse Aggregate (kg·m^−3^)	Steel Fiber (%)	SP (%)
0.16	700	200	100	1000	400	2	3

## Data Availability

The general data are included in this article. Additional data are available on request.

## Abbreviations

UHPC	Ultra-high-performance concrete	SP	Superplasticizer
PCE	Polycarboxylate superplasticizer	C_3_A	Tricalcium aluminate
SG	Sodium gluconate	XRD	X-Ray diffraction
AFt	Ettringite	SEM	Scanning electron microscopy
C_3_S	Tricalcium silicate	C-S-H	Calcium silicate hydrate
C_2_S	Dicalcium silicate	XRF	X-ray fluorescence
CH	Calcium hydroxide	C_4_AF	Tetracalcium aluminoferrite
